# Impact of Early Coherences on the Control of Ultrafast Photodissociation Reactions

**DOI:** 10.1021/acs.jpclett.3c03430

**Published:** 2024-01-31

**Authors:** Carlos G. Arcos, Alberto García-Vela, Ignacio R. Sola

**Affiliations:** † Departamento de Física Interdisciplinar, Universidad Nacional de Educación a Distancia, 28232 Las Rozas, Spain; ‡ Departamento de Química Física, Universidad Complutense de Madrid, 28040 Madrid, Spain; ¶ Instituto de Física Fundamental, Consejo Superior de Investigaciones Científicas, Serrano, 123, 28006 Madrid, Spain

## Abstract

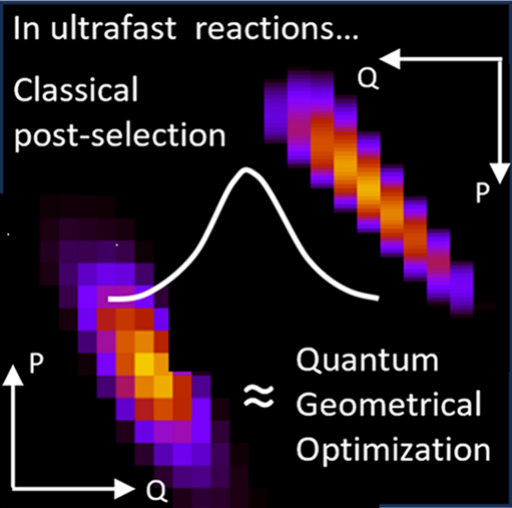

By coherent control, the yield of photodissociation reactions can be maximized, starting in a suitable superposition of vibrational states. In ultrafast processes, the interfering pathways are born from the early vibrational coherences in the ground electronic potential. We interpret their effect from a purely classical picture, in which the correlation between the initial position and momentum helps to synchronize the vibrational dynamics at the Franck–Condon window when the pulse is at its maximum intensity. In the quantum domain, we show that this localization in time and space is mediated by dynamic squeezing of the wave packet.

With the advent of ultrashort and strong laser pulses^1,2^ came a boost in the study of photodissociation reactions.^3−5^ They were primary examples in which quantum control tools were applied successfully.^6−14^ Indeed, the coherent control methods of Brumer and Shapiro^8,15,16^ and the Tannor–Rice–Kosloff scheme^17,18^ were both originally applied to photodissociation reactions with competing chemical channels, which were soon followed by more general optimal control methods.^19,20^ Challenging photochemical reactions have now been controlled using numerical schemes,^9,21,22^ or in the laboratory, by pulse shaping techniques combined with learning algorithms,^9,23,24^ but understanding the physical mechanisms behind the control remains nontrivial.^25,26^

Most of the control schemes rely on continuous laser assistance for the dynamics, for instance, to avoid conical intersections through properly timed electronic transitions,^27^ or by creating appropriate light-induced potentials with strong fields.^28−31^ Estimating the physical resources required to control the different chemical processes is a key step in finding the guiding principles that allow the control protocols. Evaluating their possible limitations with the current technology is also a necessary step. For instance, great effort is now being spent to analyze the conditions under which attochemistry,^32^ by which we mean the control of chemical reactions using attosecond pulses,^33,34^ will be possible, if it will be at all.

A cornerstone of attochemistry is the ability to manipulate the dynamics through the preparation of early electronic coherences.^35^ We can extend this rationale to femtochemistry, in which the control resources can be divided into those required to prepare the initial coherences,^36^ which are typically related to particular vibrational or bond dynamics in the ground electronic state,^37−39^ and those needed for the ignition or subsequent guidance of the dynamics. Direct photodissociation reactions can be viewed as one of the simplest chemical reactions, in which attosecond pulses (or, in general, ultrashort pulses) can be applied with success. There is a well-developed theory that can be used to choose the proper pulse parameters that maximize the yield of electronic excitation to the dissociative state, involving so-called π-pulses or chirped pulses.^14^ In principle, the control is physically limited by the bandwidth of the pump pulse.^40,41^

It is also in principle possible, although much less studied, to improve the yield of photodissociation starting from a superposition of vibrational states. This is one of the famous Brumer–Shapiro coherent control scenarios.^8,15^ In this work, we show how to generalize the scheme for any superposition state, using a variational procedure called geometrical optimization.^42−45^ Our main goal is to understand the mechanism by which this control is exerted at a fundamental level.

Exploiting the effect of interference is almost synonymous to quantum control,^46−48^ but knowing the origin and the exact role that the types of interference play is essential to properly understanding the control mechanism and its possible limitations. By analyzing the optimal superpositions in the position representation^49^ and comparing the results of quantum control with that of classical simulations, we will be able to understand why the photodissociation reaction can be controlled essentially by preparing vibrational coherences in the ground electronic state. These coherences encode the correlation between the initial position and momentum of the bond dynamics that allow maximal localization of the wave function in the Franck–Condon window, i.e., wave packet squeezing, at the time the pulse is at its peak. These are physical resources that are simple to use and to maintain, involving only nuclear degrees of freedom (no vibronic coherences are needed). As the vibrational coherences remain in the ground electronic state, they are also better protected from decoherence and decay processes than in the excited state, so we expect they can be used in the control of slower reactions. Finally, as the coherences encode correlations between positions and momenta, they can be simulated classically, so we expect that the semiclassical methods that we develop in this work may be used for the control of photodissociation reactions involving larger molecules, where many nuclear degrees of freedom are in play.

To compare in detail the results of quantum and semiclassical simulations, in this Letter we focus on the simplest system, the H_2_^+^ molecule. As described in the Supporting Information, we use a two-dimensional model (including one electronic coordinate and the internuclear distance) for the quantum results and an Ehrenfest approximation for the semiclassical simulations. It has been shown that the short-time dynamics of the photodissociation of H_2_^+^ is well reproduced by the semiclassical Ehrenfest dynamics obtained from an ensemble of 10^4^ trajectories with initial conditions chosen from the Wigner distribution of the ground vibrational state.^50^

Using transform-limited pulses in resonance with a transition between two states, the pulse area theorem^14^ shows that the population in the excited state at the end of the pulse depends on a single parameter, which is the pulse area: *A* = ∫_0_^τ^ d*tΩ*(*t*), where τ is the pulse duration and the Rabi frequency, Ω(*t*), is defined as Ω(*t*) = μ_12_ε̃(*t*)/ℏ, where ε̃(*t*) is the pulse envelope and μ_12_ the transition dipole moment. Fixing τ uniquely determines the peak pulse amplitude, ε_0_, which leads to full population inversion. The extension to electronic transitions that depend on the nuclear coordinate requires more elaborate models,^31^ but it is well established that the key to maximizing the population at the excited state is to maximize the bandwidth of the pulse, such that it spans the full absorption band. Therefore, when the pulse duration is fixed, the optimization of the pulse parameters can be reduced to a simple search of the value of ε_0_ that maximizes the yield of photodissociation, which we call a line search, as described in the Supporting Information.

Figure 1 shows the results of the line search optimization of the pulse amplitude for pulses of different durations. As expected from the pulse area theorem, the values of ε_0_ at which the probability of dissociation, *P*_0_, is maximum, ε_op_, increase to larger values for smaller values of τ. When τ = 10 fs, a maximum *P*_0_(τ, ε_op_) of 0.69 is achieved with an ε_op_ of 0.027 au, and a second (smaller) maximum is observed at twice the amplitude, manifesting the presence of Rabi oscillations. For shorter pulses, ε_op_ does not increase linearly with τ^–1^ and the maximum yields achieved are smaller, instead of larger, contradicting the expected theoretical results from the area theorem that we discussed previously. Clearly, the pulse areas are smaller than π and the yield of photodissociation stops increasing due to nonlinear effects. This occurs because the population is excited mainly to the second or higher excited states. In the regime of strong pulses that we are using, the two-electronic state approximation severely overestimates the yield of dissociation in the first excited state using short pulses and gives poor estimates of ε_op_. As shown by performing numerical simulations with different numbers of excited electronic potentials, it is important to include in the calculation at least five excited electronic states, but in all of our simulations, the yield of ionization was negligible.

**Figure 1:**
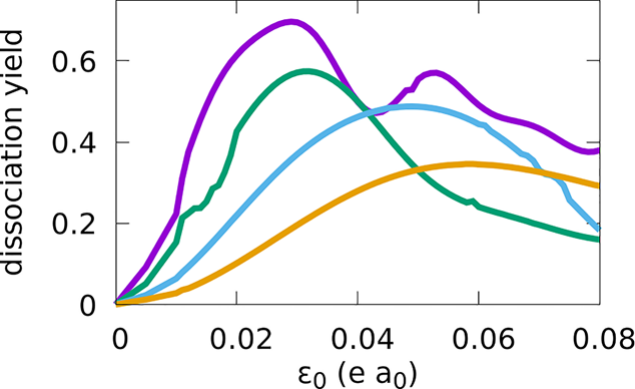
Yields of dissociation as a function of pulse peak amplitude ε_0_ for pulses with durations (τ) of 1.5 fs (orange), 2.5 fs (blue), 5 fs (green), and 10 fs (purple).

In the semiclassical approach, we use an ensemble of trajectories obtained from the Wigner function of χ_0_^*g*^(*R*). For each trajectory, we find the probability of dissociation, *P*_0_^*i*^(τ, ε_0_). Computing the average yield, *P̅*_0_(τ, ε_0_) = ∑_*i*_^*N*_*t*_^*P*_0_^*i*^(τ, ε_0_)/*N*_*t*_ as a function of ε_0_, we find the optimal pulse for the ensemble, ε̅_op_. Both ε̅_op_ and yields *P̅*_0_(τ, ε_op_) are similar to those obtained with the fully quantum approach, with changes on the order of 5%, except for the τ = 10 fs case, where *P̅*_0_(τ, ε_op_) = 0.49 and ε_op_ = 0.023 au

In direct photodissociation reactions, the reflection principle^51^ shows how the spectrum simply maps the shape of the promoted state, μ(*R*)χ_*v*_^g^(*R*), which depends on the initial vibrational state. For very short pulses, with frequencies in resonance at the Franck–Condon window, the integrated spectrum can be written in the form
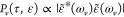
1where ε̃(ω_v_) depends on the promoted state.^52,53^ In perturbation theory, it is possible to obtain analytic formulas for eq 1 even for chirped pulses^52^ or in the presence of Stark shifts.^53^ In principle, one can optimize the field to maximize the integrated spectrum, that is, the area spanned by the entire photodissociation band, starting from any vibrational state.

However, what happens when the initial wave function is a superposition of vibrational states? According to coherent control,^8^ the integrated yield of photodissociation should have the form
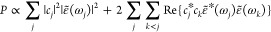
2The interference terms show that, as long as the photodissociation bands from different vibrational states overlap, one can maximize the yield by optimizing the amplitudes *c*_*j*_ of the initial state. We achieve this through a variational procedure.

We optimize the initial state to increase the yield of photodissociation for pulses of different durations, using an increasing number of vibrational states to construct the initial superposition, following the geometrical optimization method (see the Supporting Information). The results are summarized in Figure 2. The geometrical optimization performed well for all pulses. We find improvements in the yield of 36% for 10 fs pulses, 57% for 5 fs pulses, and 38% for 2.5 fs pulses. However, using the 20 lowest vibrational states, the yield of photodissociation improves by only 22% for the shortest pulse.

**Figure 2:**
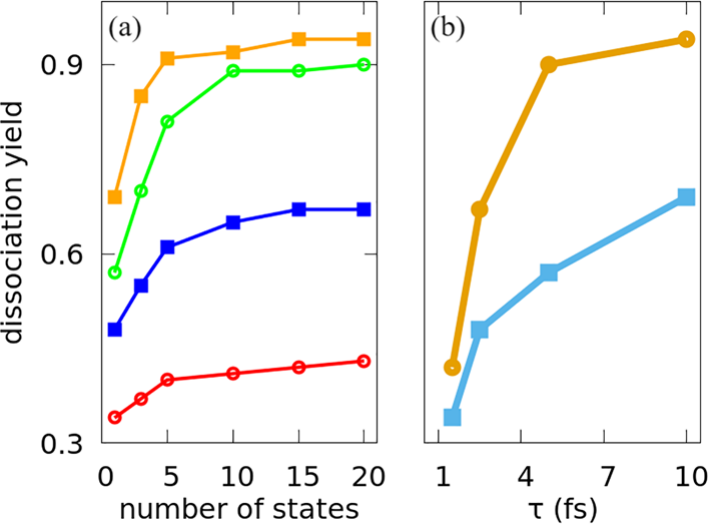
(a) Yields of dissociation from the ground state and from an optimized superposition of the *N*_b_ lowest vibrational states, as a function of *N*_b_. The pulses with durations of 1.5 fs (red), 2.5 fs (blue), 5 fs (green), and 10 fs (orange) are optimized to maximize the dissociation from the ground state. (b) Yields from the ground state (squares) and from the optimal superposition with the 20 lowest vibrational states (circles) for pulses of different durations.

Typically, there is considerable gain in *P*_op_(τ, ε_op_) when the number of states in the superposition increases up to 5 states, and then the increase is much slower with additional states, reaching its asymptotic value with an *N*_b_ of ∼20. However, for very short pulses, where the initial yield is smaller, the asymptotic results have not been reached: broader superpositions can still improve the yield.

Analyzing in detail the contributions from the different vibrational states in the optimal superpositions (Figure 3), we observe that *v* = 0 largely dominates in all cases. The population of the first excited vibrational states decays steeply (the more so, the shorter the pulse) and then exhibits some interesting patterns. It oscillates as the vibrational quantum number *v* increases (with the oscillations decaying for larger values of *v*), with peaks that shift to larger vibrational quanta as the optimal pulse becomes shorter. As eq 2 suggests, a quantum interference effect is responsible for the increase in the yield, but the origin of this interference remains to be seen. As we will show, the pattern in the populations reflects the underlying physical information, the meaning of which is mostly hidden in the energy representation.

**Figure 3:**
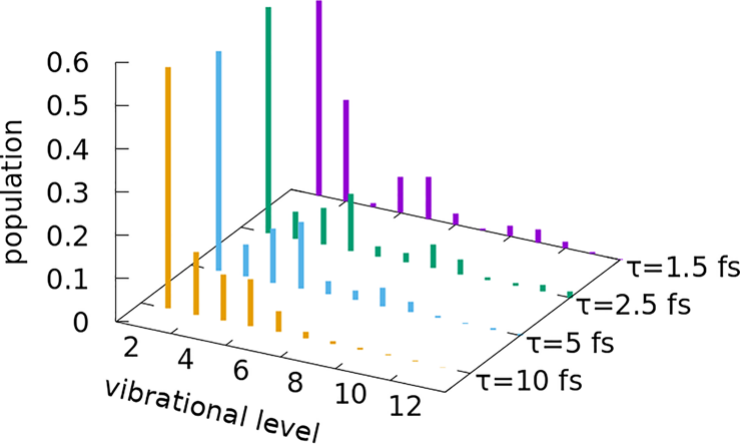
Vibrational populations of the optimized initial superposition states Ψ_op_(*z*, *R*, 0) for pulses of different durations. The populations oscillate with the vibrational quantum number, especially for shorter pulses.

Because the nuclear degrees of freedom are not quantized, we need to develop an alternative procedure to optimize the initial state in the Ehrenfest dynamics. In this work, we do so by a process of distillation of initial conditions, which we call postselection of trajectories.

We first need to decide from which initial distribution of nuclear degrees of freedom we calculate *P̅*(τ, ε̅_op_), which is in some way comparable to the set of vibrational states included in Ψ_op_. We have performed calculations starting from a uniform distribution of [*R*^*i*^(0), *p*^*i*^(0)] for all of the classically accessible phase space in the energy range spanned by the set of vibrational states up to an *N*_b_ of 20. We have also performed calculations starting from the Wigner function of χ_0_^*g*^, which is a Gaussian distribution with standard deviations (in atomic units) *ΔR* = 0.2305, *Δp* = 2.1692, and with wider Gaussian distributions in both dimensions. As we comment below, in view of the postselection that we follow to “optimize” the initial state, the choice of the initial distribution does not qualitatively change the results. We show below the results obtained from the Gaussian distribution of the ground vibrational state. For each initial condition [*R*^*i*^(0), *P*^*i*^(0)], we evaluate the yield of dissociation *P*_0_^*i*^(τ, ε̅_op_) (eq 7 in the Supporting Information) in the color map of Figure 4.

**Figure 4:**
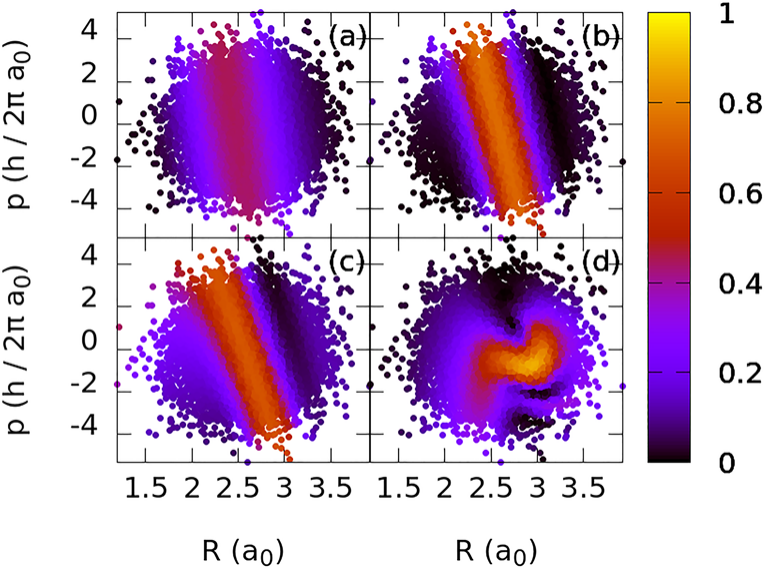
Yields of photodissociation as a function of the initial distribution after application of the optimal pulse ε̅_op_, for different pulse durations (τ): (a) 1.5, (b) 2.5, (c) 5, and (d) 10 fs. The initial distribution is the Wigner function of χ_0_^*g*^. Higher yields appear on straplike regions of the phase space.

As the phase-space distribution of *P*_0_^*i*^(τ, ε̅_op_) shows, the initial momentum and the initial internuclear distance must be linearly anticorrelated to maximize the probability of excitation, leading to the strips of higher yields in the map. For small values of *R*^*i*^(0) (bond compression), one needs large positive momentum *p*^*i*^(0) values to achieve a high *P*_0_^*i*^(τ, ε̅_op_), whereas large negative momenta *p*^*i*^(0) are needed for large values of *R*^*i*^(0), when the bond is initially stretched.

The reason for this correlation is clear. The population is mainly transferred to the excited dissociative state in the Franck–Condon window, which, for the chosen frequency of the pulse, is close to the equilibrium bond distance. For a pulse shorter than a vibrational period (*T* ≈ 16.7 fs), if initially stretched, the bond must be shortening at a certain speed [given by *p*^*i*^(0)], to reach the equilibrium bond distance at the maximum intensity, thereby maximizing the population transfer. On the contrary, if the bond is initially compressed, it must be stretching. For shorter pulses, the required momentum to reach the equilibrium distance at the maximum intensity must be larger. Hence, the largest values of *P*^*i*^(τ, ε̅_op_) show a more tilted distribution in the phase space for shorter pulses. Only for longer pulses (e.g., τ = 10 fs) is there sufficient time for the bond to reach the equilibrium bond distance at the maximum intensity of the pulse for different values of *p*^*i*^(0), as the bond can stretch and then compress in more than one period. Hence, the distribution in phase space of *P*^*i*^(τ, ε̅_op_) in Figure 4d shows no clear tilt. On the other hand, because the Franck–Condon window is narrower using longer pulses, since the smaller pulse bandwidth imposes more stringent resonance conditions, fewer initial conditions can be used to obtain *P*^*i*^(τ, ε̅_op_) in such a case.

The classical equivalent to the geometrical optimization process that we follow in this work is the selection of the set of initial conditions such that the semiclassical results equal those of the geometrical optimization. To do so, we order the trajectories from higher to lower yields and obtain average yields *P̅*_op_(τ, ε̅_op_) = ∑_*i*_^*N*_sel_^*P*_0_^*i*^(τ; ε̅_op_)/*N*_sel_. The cutoff of the initial conditions, *N*_sel_, is chosen such that *P̅*_op_(τ, ε̅_op_) = *P*_op_(τ, ε_op_). In principle, one could select a very small subset of trajectories to obtain even higher average yields, particularly for long pulses, as there are initial conditions that lead to almost full dissociation, but our procedure allows for an unbiased comparison of the classical and optimal initial distributions.

The “optimal” phase-space distributions extract from Figure 4 those configurations that give large values of *P*^*i*^(τ, ε_op_). Therefore, the distributions exhibit a distinctive linear correlation between the initial positions and the initial momentum, which is commonly termed chirp, β = d*p*^*i*^(0)/d*R*^*i*^(0) . For the different pulse durations, we obtain β(τ = 1.5) = −32ℏ/*a*_0_^2^, β(τ = 2.5) = −14ℏ/*a*_0_^2^, β(τ = 5) = −5.9ℏ/*a*_0_^2^, and β(τ = 10) = −4.2ℏ/*a*_0_^2^. The results are shown in Figure 5.

**Figure 5:**
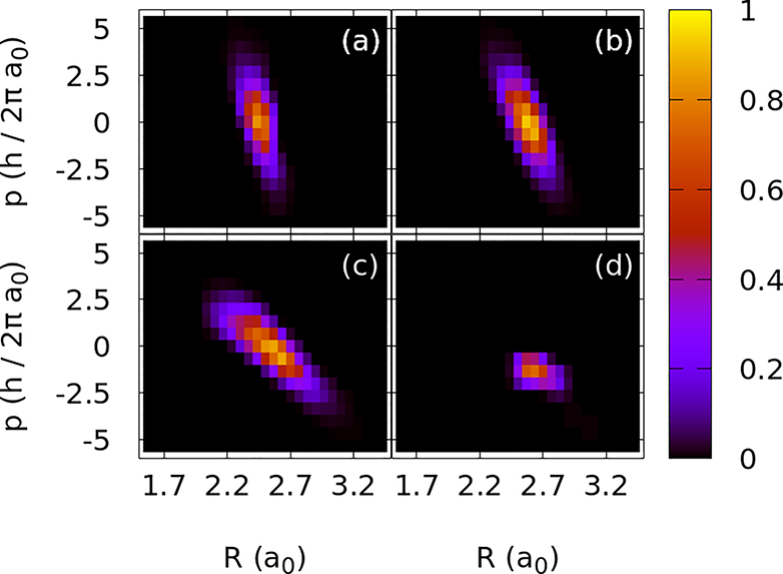
Optimal initial distributions in phase space obtained by selecting the initial conditions such that the average yield for the classical ensemble of trajectories equals the quantum yield obtained after the geometrical optimization, for pulses with durations (τ) of (a) 1.5, (b) 2.5, (c) 5, and (d) 10 fs. The phase-space distributions are chirped, showing a distinctive correlation of the initial nuclear position and momentum for those trajectories that maximize the yield of photodissociation.

Starting from wider initial distributions in phase space leads to essentially the same type of optimal distributions, but adding the contribution from trajectories that start from values of *R*^*i*^(0) more distant from *R*_0_, with corresponding larger (positive or negative) momenta *P*^*i*^(0). However, we would require many trajectories to fully characterize this distribution (because they occupy a larger volume of phase space) without biasing the generation of random conditions.

Quantum correlations between the position and momentum can be observed by calculating the Wigner distribution of the initial optimal wave functions, Ψ_op_, that are shown in Figure 6. The momentum–position correlations are imprinted as coherences in Ψ_op_, as the phase (momentum) must depend on the position of the wave function. These correlations have a counterpart in the patterns observed for the optimal populations (and phases) in the energy representation (Figure 3).

**Figure 6:**
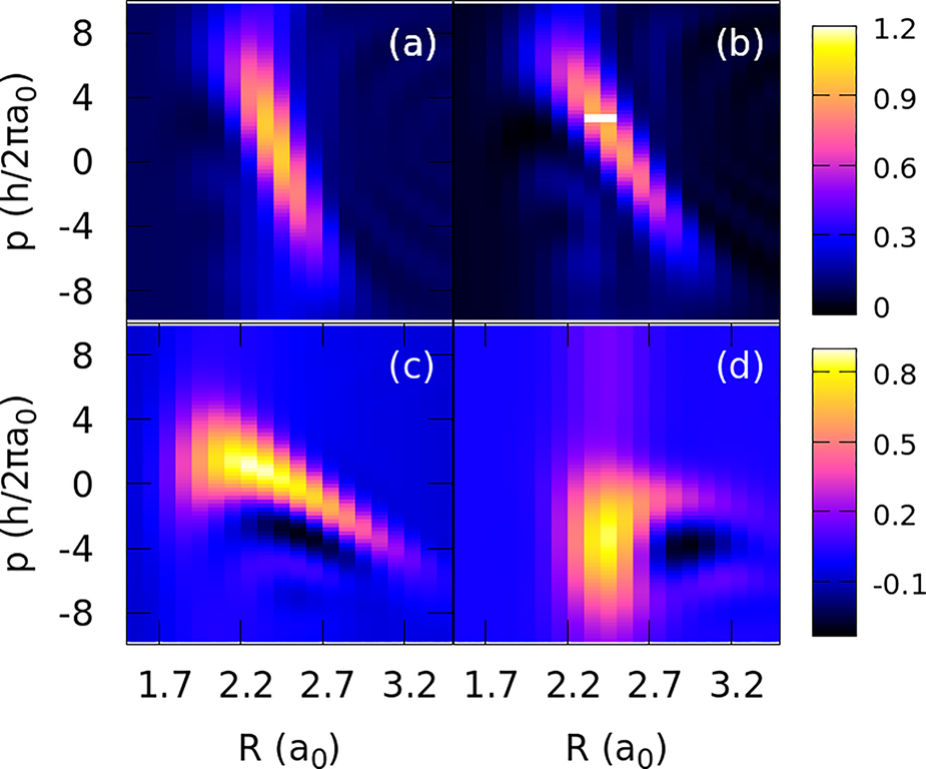
Wigner functions of optimized initial wave functions Ψ_op_ for pulses with durations (τ) (a) 1.5, (b) 2.5, (c) 5, and (d) 10 fs. The distributions are similar to those obtained by the semiclassical optimization.

The Wigner functions are negatively chirped for all pulse durations except for τ = 10 fs, which has no clear chirping. An approximate estimate of the chirp gives β(τ = 1.5) = −28ℏ/*a*_0_^2^, β(τ = 2.5) = −14ℏ/*a*_0_^2^, and β(τ = 5) = −6.5ℏ/*a*_0_^2^. In the latter case, one can observe some nonlinear contribution to the chirp as well as some regions where the Wigner distribution is negative, for which the distribution has no classical interpretation. This also occurs for a τ of 10 fs, for which we did not try to determine β. Overall, the initial functions for the short pulses resemble the classical “optimal” phase-space distributions but are slightly shifted to shorter bond distances. For longer pulses, the differences between the classical and quantum distributions are more prominent.

This feature of the Wigner functions suggests a physical mechanism for the population transfer analogous to what is observed in the classical distributions. However, unlike that in the postselection of trajectories, a quantum state always occupies a certain volume in phase space from the Heisenberg principle, so the initial chirping provokes dynamical squeezing of the wave packet.^54,55^ The spatially wide (chirped) initial wave function, quite stretched beyond χ_0_^*g*^(*R*), is prepared in such a way that the function becomes dynamically compressed in the Franck–Condon region at the time the pulse is at its peak, maximizing the excitation probability. This effect can be observed in Figure 7, where we show snapshots of the wave packet at times *t* = 0 and *t* = τ/2 (the maximum intensity of the pulse) for different pulse durations. At τ/2, the relative spreads in the position of the wave packet, measured with respect to the width of χ_0_^*g*^, defined as γ(τ) = *Δψ*(τ/2)/*Δχ*_0_^*g*^, are γ(1.5) = 0.51, γ(2.5) = 0.44, γ(5) = 0.48, and γ(10) = 0.49.

**Figure 7:**
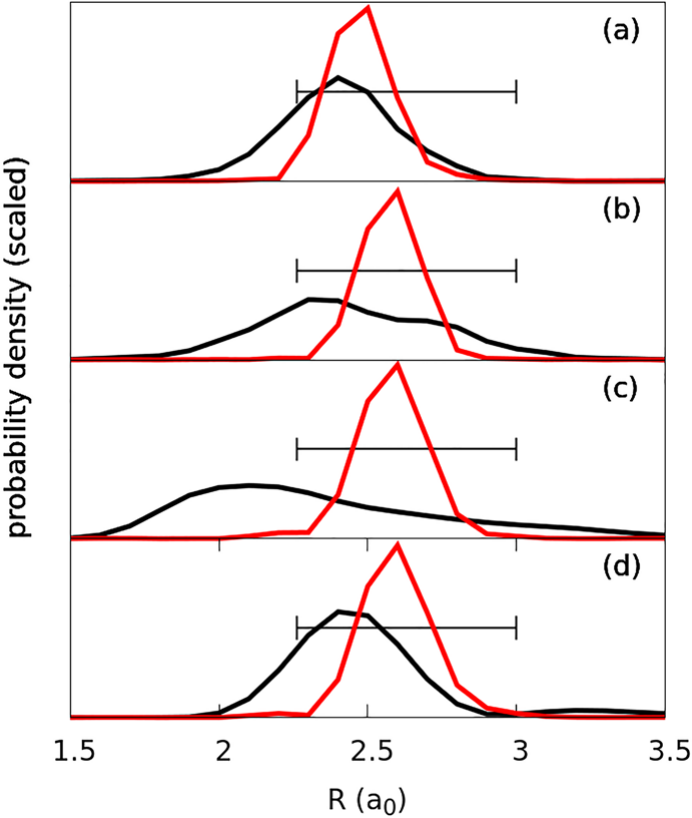
Probability densities of the optimal wave functions at the initial time (black line) and at the maximum intensity (red line) for pulses with durations (τ) of (a) 1.5, (b) 2.5, (c) 5, and (d) 10 fs. The segment shows the full width at half-maximum of χ_0_^*g*^. The dynamics shows squeezing in the bond length of the molecule at the Franck–Condon window, which occurs at the time the pulses are at their peak, maximizing the rate of electronic excitation.

For very short pulses, the chirp must be very large. The wave function is broader in momentum than in position. For longer pulses (e.g., τ = 5 fs), the opposite occurs. As the wave function extends over a larger set of initial bond distances, one must take into account the anharmonicity of the potential to maximize the localization of the packet at the optimal time. Thus, there are nonlinear contributions to the chirp. How can one achieve such an effect when τ = 10 fs, where no significant chirp is observed? The squeezing can also be achieved if the wave function is initially compressed or stretched. In a harmonic potential, the wave function will breathe at a period twice ω_*v*_ (the fundamental harmonic frequency, which is approximately 16.7 fs in our case).^54,55^ Hence, after starting in a squeezed state, the system will reach another squeezed state after ∼8.3 fs, slightly after the peak of the pulse.

Combining simple techniques of optical and geometrical control, we have shown that one can maximize the yield of ultrafast photodissociation reactions, which occur in a very few femtoseconds. A key step in the control of these ultrafast reactions lies in the preparation of the initial state. We examined the impact of the early coherences in the initial wave function. From a wider perspective, one can understand their role as favoring constructively interfering pathways leading to the product as a generalization of one of the elegant coherent control schemes of Brumer and Shapiro.

To fully understand how the interference takes place, one must analyze the dynamics in the proper representation, which, in this case, is the spatial domain. Then we observe that the interference takes place solely on the ground electronic potential energy curve by means of dynamical squeezing of the wave packet. The process leads to maximally exploiting the Franck–Condon window at the time the pulse is at its maximum. The results of trajectory simulations in the Ehrenfest approximation point to a similar mechanism that can be exploited (without interference) in the classical regime. Indeed, in analogy with the geometrical optimization, we have developed a semiclassical procedure that finds the “optimal” initial distributions in phase space, which closely resemble the Wigner functions of the optimal initial states and give similar yields, at least for sufficiently short pulses.

In this work, we have focused on a very simple model, but the principles of the method can be easily extrapolated to polyatomic molecules. When several vibrational modes are involved, it is expected that ultrafast photodissociation reactions will rely on choosing a proper direction along which the initially created wave packet must move before the pulse hits the molecule, which should maximize the probability of finding the packet at the optimal time in the Franck–Condon window. In many molecules, where the transition moment is largest in a given local mode, this mode should dominate the reaction so that an effective one-dimensional Hamiltonian could prove to be sufficient for modeling the process. In other cases, where many atomic motions are involved, semiclassical approximations may be unavoidable. We have shown that a family of solutions exists in the classical regime where the correlation between the initial elongation and the momenta of the bonds accounts for most of the quantum coherences involved in the control processes. Even complex reactions may require the avoidance of regions on the excited potential (e.g., conical intersections, etc.). One could conceive of preparing the initial state as a superposition involving vibronic wave packets in different electronic states, such as those that are now becoming possible with attosecond pulses. In this case, a fully classical treatment of the motion of the electron and the nuclei as performed in ref (56) could reveal information about correlations on the electronic position and momentum, in addition to those of the bond dynamics, necessary to control the reaction. In principle, the techniques developed in this work could be extended to more complex scenarios. Indeed, the control of early electronic and vibrational coherences may be a key step in attochemistry, and the evaluation of all of its possibilities will be essential for foreseeing its possible impact in different photochemical processes. The geometrical optimization is a very useful procedure that separates how the different physical resources (photons, ground state coherences, and excited state coherences) are used to control a given reaction, but ultimately, the initial state must be prepared by a proper laser protocol. In our case, short and strong infrared pulses (or impulsive Raman pulses) could be needed in addition to the pump pulse. Then the time delay between the pulses as well as their respective intensities and durations will become the essential control parameters. Work along these lines is in progress.

## Supporting Information Available

Model Hamiltonian, Ehrenfest model, optimal pulses, geometrical optimization, and additional references (PDF)
