# Supplementary material for: Impact of Early Coherences on the Control of Ultrafast Photodissociation Reactions
Source: J Phys Chem Lett. 2024 Jan 31;15(5):1442–8. doi: 10.1021/acs.jpclett.3c03430 (PMC10860130; doi:10.1021/acs.jpclett.3c03430)
Supplement: Supplementary file 1 — jz3c03430_si_001.pdf [file jz3c03430_si_001.pdf]

Supplementary information: The Impact of Early Coherences in the Control of Ultrafast Photodissociation Reactions

Carlos G. Arcos,[†] Alberto García-Vela,[‡] and Ignacio R. Sola[*,¶]

†*Dept. Física Interdisciplinar, Universidad Nacional de Educación a Distancia, 28232 Las Rozas, Spain*

‡*Instituto de Física Fundamental, Consejo Superior de Investigaciones Científicas, Serrano, 123, 28006 Madrid, Spain*

¶*Departamento de Química Física, Universidad Complutense de Madrid, 28040 Madrid, Spain*

E-mail: isolarei@ucm.es

Model Hamiltonian

Reduced dimensional Hamiltonians allow the study of the photodissociation and ionization of H_2^+ in the presence of strong and short pulses with qualitative agreement with experiment.[1,2] We use a simple one-dimensional model where the electron moves along the bond axis, interacting with the nuclei through the well-known soft-core Coulomb potential,[3,4]

$$V_{SC}(z,R) = -\frac{1}{\sqrt{((z+R/2)^2+1}} - \frac{1}{\sqrt{((z-R/2)^2+1}} + \frac{1}{R}\ , \tag{1}$$

where z is the electron coordinate and R the internuclear distance, and solve the TDSE (time-dependent Schrödinger equation) for one electronic plus one nuclear degree of freedom:

$$i\frac{d}{dt}\Psi(z,R,t) = -\frac{1}{2}\frac{\partial^2}{\partial z^2}\Psi(z,R,t) - \frac{1}{M}\frac{\partial^2}{\partial R^2}\Psi(z,R,t) + [V_{SC}(z,R) + \varepsilon(t)z]\Psi(z,R,t) \tag{2}$$

(where M is the Hydrogen mass), using a split-operator method[5,6] in a grid of 1024 points in the z coordinate, ranging between $-35\ a_0$ to $35\ a_0$, and 256 points in the R coordinate, ranging between $0.5\ a_0$ and $9.9\ a_0$. Imaginary absorbing barriers near the grid limits are included to avoid unphysical processes at the edges of the grid. We use sine square shaped pulses, $\varepsilon(t) = \varepsilon_0 \sin^2(\pi t/\tau)\sin(\omega t)$, defined for $t \in [0, \tau]$.

The initial vibronic states are built as

$$\Psi_v(z,R,0) = \chi_v^g(R)\phi^g(z;R) \tag{3}$$

where the electronic and vibrational eigenstates are obtained using the Fourier Grid Hamiltonian method[7] (FGH). We first apply the FGH to solve for the eigenstates of the electronic Hamiltonian at each fixed internuclear distance, evaluated at every nuclear grid point R_α, obtaining the ground and excited electronic potential energy curves $V_g(R)$, $V_e(R)$ and the corresponding electronic states $\phi^g(z;R)$ and $\phi^e(z;R)$. Then we obtain the vibrational eigenfunctions of the ground state diagonalizing the electronic Hamiltonian on the grid, $\hat{H}^g = -(1/M)d^2/dR^2 + V_g(R)$, using the same FGH method.

Ehrenfest model

Although it is well known that a trajectory in the semiclassical Ehrenfest approach cannot give the correct asymptotic dynamics whenever the electronic state is a superposition of two different electronic states, an ensemble of trajectories mimics the behavior of the dissociating wave packet in the short-time scale dynamics.[1] On the other hand, for strong pulses, the dynamics during

the pulse interaction is typically not well reproduced by surface-hopping techniques,[8–10] unless one uses a dressed-state representation.[11] We follow the approach in,[1,12] by which we generate an ensemble of 10^4 initial conditions $\left(R^i(0), p^i(0)\right)$ for the nuclear degrees of freedom from the Wigner distribution of $\chi_0^g(R)$, and use $R^i(t)$ to calculate $V_{SC}\left(z, R^i(t)\right)$. The initial state is defined as $\psi^i(z,0) = \phi^g(z; R_\beta)$, where R_β is the closest nuclear grid point to the sampled initial configuration, $R^i(0)$. The electronic wave function for the trajectory i, $\psi^i(z,t)$, evolves through the TDSE

$$i\frac{d}{dt}\psi^i(z,t) = -\frac{1}{2}\frac{d^2}{dz^2}\psi^i(z,t) + \left[V_{SC}(z, R(t)) + \varepsilon(t)z\right]\psi^i(z,t) \tag{4}$$

and the nuclear positions follow the average force,

$$\frac{d^2R^i(t)}{dt^2} = -\frac{1}{M}\left\langle \psi^i(z,t)\right| \frac{\partial V_{sc}\left(z, R^i(t)\right)}{\partial R}\left|\psi^i(z,t)\right\rangle \ . \tag{5}$$

Newton's equations are solved with a fourth-order Runge-Kutta integrator.[13] All observables are finally averaged over the N_t trajectories.

Optimal pulses

We find the optimal pulse amplitude for fixed pulse duration τ, by simply performing simulations varying ε_0 and choosing the parameter that gives higher yields, ε_{op}. We call this single-parameter control method, a line-search approach. Because we use large nuclear grids and very short pulses, we can neglect the loss of population through the imaginary barrier and the dissociation probability is simply calculated as the norm in the excited electronic state,

$$P_0(\tau, \varepsilon_0) = \left|\langle \phi^e(z;R)|\Psi_0(z,R,\tau)\rangle_{z,R}\right|^2 \tag{6}$$

where $\Psi_0(z,R,\tau)$ is obtained solving the TDSE with a pulse of peak amplitude ε_0 starting from the ground vibronic state, $\chi_0^g\phi^g$. The subindex in the bracket indicates the coordinates over which we

perform the integral.

In the semiclassical model, the probability is calculated for each trajectory i as,

$$P_0^i(\tau,\varepsilon_0) = \left| \langle \phi^e(z;R_\alpha) | \psi_0^i(z,\tau) \rangle_z \right|^2 \tag{7}$$

where R_α is the closest nuclear grid point to the internuclear distance for the trajectory i at the end of the pulse, $R^i(\tau)$, and $\psi_0^i(z,\tau)$ is obtained solving the TDSE using the semiclassical Ehrenfest method. Then, $P_0^i(\tau,\varepsilon_0)$ are averaged over 10^4 trajectories calculated from the different $R^i(0)$ (and the initial momentum, $p^i(0)$) obtained from the Wigner distribution[14] of χ_0^g, and the result is called $\bar{P}_0(\tau,\varepsilon_0)$.

Geometrical Optimization

The geometrical optimization is the procedure by which the yield is maximized with respect to any initial wave function $\psi(z,R,0)$, constrained such that its norm is conserved. This is a Rayleigh-Ritz problem by which the optimal initial wave functions are the eigenfunctions of the yield operator, $\hat{P}_H(\tau)$, which is a projection operator in the Heisenberg picture.[15–17] For the photodissociation reaction, from Eq.(6)

$$\hat{P}_H(\tau) = \hat{U}^{-1}(\tau;\varepsilon(t))\, |\phi^e(z;R)\rangle\langle\phi^e(z;R)|\, \hat{U}(\tau;\varepsilon(t)) \ . \tag{8}$$

Useful solutions of this equation can be obtained limiting the shape of the accepted initial optimal wave functions as superpositions of a set of vibrational eigenfunctions of the ground electronic state,

$$\Psi_{\mathrm{op}}(z,R,0) = \sum_v^{N_b} c_v(0)\chi_v^g(R)\phi^g(z;R) \tag{9}$$

with optimized amplitudes $c_v(0)$, for different values of N_b. Then Eq.(8) becomes an eigenvector equation of the matrix S, with elements[17]

$$S_{v',v} = \langle \Psi_{v'}(z,R,\tau) | \phi^e(z;R) \rangle_{z,R} \langle \phi^e(z;R) | \Psi_v(z,R,\tau) \rangle_{z,R} \quad (10)$$

where $\Psi_v(z,R,\tau)$ is obtained solving the TDSE from the initial state $\Psi_v(z,R,0) = \chi_v^g(R)\phi^g(z;R)$. The diagonal elements of matrix S give the photodissociation yields obtained from the different starting vibrational eigenstates of the ground state. Its largest eigenvalue, $P_{\rm op}(\tau,\varepsilon_0)$, gives the maximum possible photodissociation yield for an initial wave function of the form of Eq.(9) driven by the field $\varepsilon_{\rm op}(t)$, which will be chosen as the field that maximizes the yield from $\Psi_0(z,R,\tau)$, using the line search approach.

To find the optimal superposition, we solve the TDSE starting from different vibrational eigenstates of the ground electronic state, $\Psi_v(z,R,0)$ and compute $s_v = \langle \phi^e(z;R) | \Psi_v(z,R,\tau) \rangle_{z,R}$. Then the matrix S is constructed and its eigenvalues and eigenvectors are obtained by Jacobi diagonalization.[13]

References

(1) Chang, B. Y.; Shin, S.; Malinovsky, V. S.; Sola, I. R. Grid-based Ehrenfest model to study electron–nuclear processes. *J. Phys. Chem. A* **2019**, *123*, 7171–7176.

(2) Carrasco, S.; Rogan, J.; Valdivia, J. A.; Sola, I. R. Anti-alignment driven dynamics in the excited states of molecules under strong fields. *Phys. Chem. Chem. Phys.* **2021**, *23*, 1936–1942.

(3) Javanainen, J.; Eberly, J. H.; Su, Q. Numerical simulations of multiphoton ionization and above-threshold electron spectra. *Phys. Rev. A* **1988**, *38*, 3430–3446.

(4) Kulander, K.; Mies, F.; Schafer, K. Model for studies of laser-induced nonlinear processes in molecules. *Phys. Rev. A* **1996**, *53*, 2562.

(5) Kosloff, R. Time-dependent quantum-mechanical methods for molecular dynamics. *J. Phys. Chem.* **1988**, *92*, 2087–2100.

(6) Kosloff, R. Propagation methods for quantum molecular dynamics. *Ann. Rev. Phys. Chem.* **1994**, *45*, 145–178.

(7) Marston, C. C.; Balint-Kurti, G. G. The Fourier grid Hamiltonian method for bound state eigenvalues and eigenfunctions. *J. Chem. Phys.* **1989**, *91*, 3571–3576.

(8) Mitrić, R.; Petersen, J.; Bonačić-Kouteckỳ, V. Laser-field-induced surface-hopping method for the simulation and control of ultrafast photodynamics. *Phys. Rev. A* **2009**, *79*, 053416.

(9) Marquetand, P.; Richter, M.; González-Vázquez, J.; Sola, I.; González, L. Nonadiabatic ab initio molecular dynamics including spin–orbit coupling and laser fields. *Faraday Discuss.* **2011**, *153*, 261–273.

(10) Richter, M.; Marquetand, P.; González-Vázquez, J.; Sola, I.; González, L. SHARC: ab initio molecular dynamics with surface hopping in the adiabatic representation including arbitrary couplings. *J. Chem. Theory Comput.* **2011**, *7*, 1253–1258.

(11) Bajo, J. J.; Gonzalez-Vazquez, J.; Sola, I. R.; Santamaria, J.; Richter, M.; Marquetand, P.; González, L. Mixed quantum-classical dynamics in the adiabatic representation to simulate molecules driven by strong laser pulses. *J. Phys. Chem. A* **2012**, *116*, 2800–2807.

(12) Chang, B. Y.; Shin, S.; González-Vázquez, J.; Martín, F.; Malinovsky, V. S.; Sola, I. R. Control defeasance by anti-alignment in the excited state. *Phys. Chem. Chem. Phys.* **2019**, *21*, 23620–23625.

(13) Vetterling, W. T.; Press, W. H. *Numerical recipes in Fortran: the art of scientific computing*; Cambridge University Press, 1992; Vol. 1.

(14) Weinbub, J.; Ferry, D. Recent advances in Wigner function approaches. *Appl. Phys. Rev.* **2018**, *5*, 041104.

(15) Chang, B. Y.; Shin, S.; Sola, I. R. Ultrafast population inversion without the strong field catch: the parallel transfer. *J. Phys. Chem. Lett.* **2015**, *6*, 1724–1728.

(16) Chang, B. Y.; Shin, S.; Sola, I. R. State-selective excitation of quantum systems via geometrical optimization. *J. Chem. Theory Comput.* **2015**, *11*, 4005–4010.

(17) Sola, I. R.; Chang, B. Y.; Malinovskaya, S. A.; Malinovsky, V. S. *Adv. At. Mol. Opt. Phy.*; Elsevier, 2018; Vol. 67; pp 151–256.
